# Morphological and molecular study of Symphyla from Colombia

**DOI:** 10.3897/zookeys.484.8363

**Published:** 2015-03-09

**Authors:** Diego A. Salazar-Moncada, Jaime Calle-Osorno, Freddy Ruiz-Lopez

**Affiliations:** 1 Grupo de Bio-control y Microbiología aplicada (BIOMA), Instituto de Biología, Facultad de Ciencias Exactas y Naturales, Universidad de Antioquia. Calle 67 No. 53-108. Medellín, Colombia; 2 Programa de Estudio y Control de Enfermedades Tropicales – PECET, Universidad de Antioquia. Lab. 632. Calle 62 No. 52-59. Medellín, Colombia

**Keywords:** *Scutigerella immaculata*, Colombia, *COI* barcode, ITS2, morphology

## Abstract

The symphylans are a poorly studied group. In Colombia the number of symphylan species is unknown with only *Scutigerella immaculata* (Symphyla: Scutigerellidae) being reported previously. The aim of this research was to collect and identify the symphylan pests of flower crops in Colombia. Morphological descriptions showed that our specimens shared more than one of the characters that define different genera within Scutigerellidae. The *COI* barcode haplotype showed interspecific level genetic divergence with *Scutigerella causeyae* (at least 23%) and *Hanseniella* sp. (22%). Furthermore, our Colombian symphylans shared the same *COI* haplotype as some Symphyla found in Cameroon indicating a wide geographical distribution of this taxon. Our results suggest the presence of a new genus or subgenus in the class Symphyla.

## Introduction

The symphylans (Arthropoda: Symphyla) are ancestral arthropods dating back to the early Silurian approximately 430 million years ago (Edgecombe 2004, Shear and Edgecombe 2010). Symphylans are a phylogenetic enigma within arthropods as they have been proposed as sister taxa to different groups (Domínguez 2009). Symphyla is comprised of two families: Scutigerellidae (five genera and approximately 128 species) and Scolopendrellidae (nine genera and approximately 73 species) (Domínguez 2009). Symphylan species are morphologically determined mainly based on the chaetotaxy of the head, antennae size and shape of the scuta margins (Domínguez 1992, Edwards 1959a, b, Scheller 1961).

Only two genera in the family Scutigerellidae are considered to be pests in a wide range of crops: *Scutigerella* Ryder, 1882 and *Hanseniella* Bagnal, 1913 (Michelbacher 1938). *Scutigerella immaculata* Newport, 1845 is the only reported symphylan in Colombia where it is regarded as a pest of pineapple (Agredo 1988) and flower crops (Duran 1982, Navarro and Gaviria 2001). However, in these reports the authors did not describe how they identified *Scutigerella immaculata*. Questions are raised regarding the presence of *Scutigerella immaculata* in tropical Colombia. Domínguez (2009) only reports *Scutigerella* genus in northern temperate zones. In northern Brazil, bordering Colombia and Peru, de Morais and da Silva (2009) report the presence of *Hanseniella* and *Symphylella* (Scolopendrellidae). The distribution of the family Scutigerellidae is: *Scutigerella* mainly in northern temperate zones; *Hanseniella* in tropical and warm temperate zones; *Millotellina* in Africa, Madagascar, Réunion, Sri Lanka, New Guinea and Australia; *Scolopendrelloides* in South-East Asia and Australia; and *Scopoliella* in North America only (Domínguez 2009).

Mitochondrial DNA *Cytochrome Oxidase I* (*COI*) barcode region (Hebert et al. 2003, Smith et al. 2005) and the ribosomal nuclear Internal Transcribed Spacer 2 (ITS2) are used as molecular markers for arthropod species identification (Hebert et al. 2003, Ruiz et al. 2005, Wiemers et al. 2009). Barcoding is a fast and accurate method for species delimitation using the Kimura Two-Parameter model (K2P) (Padial and De la Riva 2007). There are few reports using these molecular makers in symphylans (Mallatt et al. 2004, Podsiadlowski et al. 2007, Spelda et al. 2011, Stoev et al. 2010, 2013) and none characterising Colombian symphylans.

Symphylan pests in Colombia are commonly identified as *Scutigerella immaculata* by the presence of a single morphological feature, a U-shape groove in the scuta of the last abdominal segment. The aim of this study was to capture symphylans in two departments of Colombia and describe these using multiple morphological characters and molecular markers.

## Methods

### Symphylan collection and examination

Symphylans were collected from two flower companies: Flores Esmeralda S.A.S C.I. in Antioquia (6°1'0"N, 75°25'0"W, 2180 m.a.s.l.) and Flexport and CIA.S.A.C.I. in Cundinamarca (4°45'4.10"N, 74°13'30.87"W, 2548 m.a.s.l.). Symphylan collection used a modified method of Umble et al. (2006); beet slices instead of potato baits covered with black plastic to block the passage of light were set overnight for 12 hours on flowerbeds. The next morning, the symphylans were collected from the beets and soil around the baits and transported in Petri dishes – 20 individuals per dish, each dish 9 cm in diameter, containing 17 g of soil (previously sterilized at 121 °C) and beet as a food source – to the Bio-control and Microbiology Laboratory (BIOMA), University of Antioquia, Medellín, Colombia. Symphylans were identified by morphology (N = 30) using the descriptions and keys of Domínguez (2009, 2010), Halliday (2004), and Naumann and Scheller (1977). A total of 15 specimens from Antioquia (N = 10) and Cundinamarca (N = 5) were imaged using the Scanning Electron Microscope (SEM, Hitachi S-510) methodology of A. Acevedo (unpublished). In short, specimens were first fixed in 2% glutaraldehyde and then subsequently fixed in 1% osmium tetraxide. Each sample was dehydrated in up to 100% ethanol, critical-point dried and sputter coated with gold. Vouchers specimens are stored in BIOMA laboratory, University of Antioquia.

### Molecular characterisation

DNAs of ten symphylans from Antioquia were extracted using DNeasy Blood and Tissue Kit (QIAgen®, USA). The *COI* barcode region was amplified by polymerase chain reaction (PCR) using the primers developed by Folmer et al. (1994) and following the protocol of Ruiz et al. (2010). The rDNA ITS2 PCR was carried out using the primers of Collins and Paskewitz (1996) following the protocol of Linton et al. (2001).

Bi-directional sequencing used the Big Dye Terminator Kit® on an ABI3730 automated sequencer (PE Applied BioSystems, Warrington, England). Raw sequence chromatograms were edited using Sequencher™ v. 4.8 (Genes Codes Corporation, Ann Arbor, MI), aligned automatically in MAFFT v. 7 (ITS2) (Katoh et al. 2002) or manually (*COI*) using MacClade v. 4.06 (Maddison and Maddison 2003). Sequence similarities were compared with those available (October 14, 2014) in GenBank using Basic Local Alignment Search Tool (BLAST) (http://blast.ncbi.nlm.nih.gov/Blast.cgi) and Barcoding of Life Data Systems (BOLD Systems) (http://www.barcodinglife.com/).

## Results

A total of 210 symphylans were collected from Antioquia (N = 180) and Cundinamarca (N = 30) and some were used for morphological and molecular studies.

### Morphology

**Figure 1.**
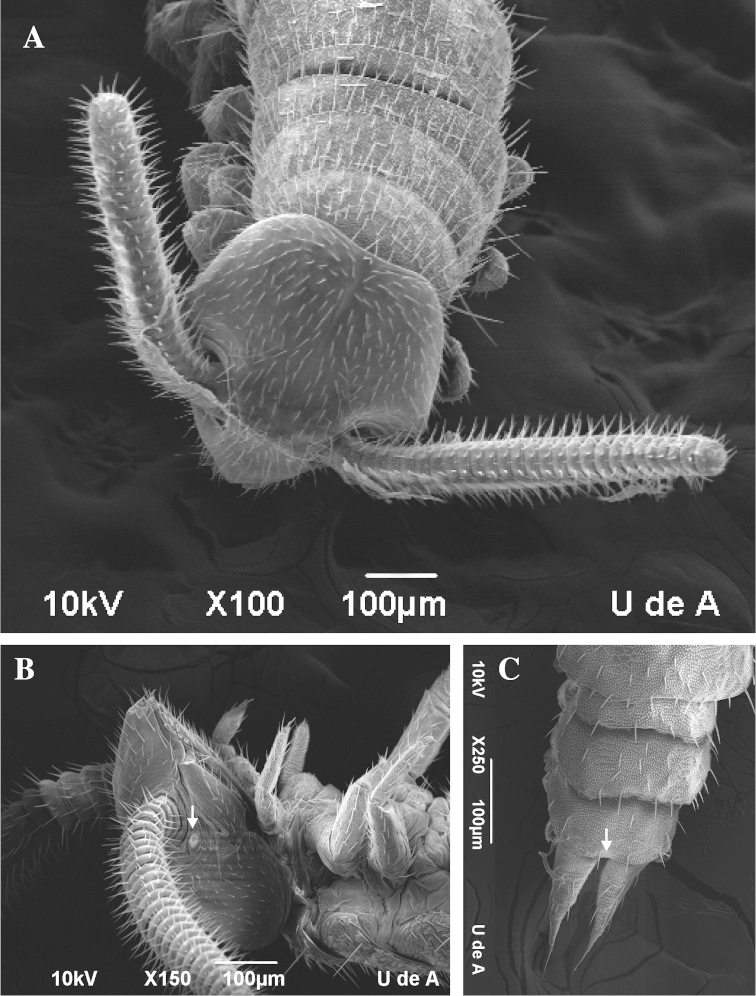
Colombian symphylan. **A** Heart-shaped head and antennae **B** Tömosvary organ (arrow) **C** Last scuta margin with a U-shaped groove (arrow).

**Figure 2.**
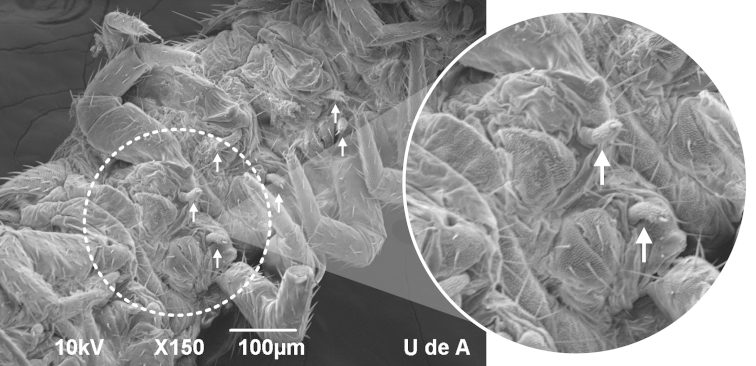
Ventral view of a Colombian symphylan. Presence of sternal appendages behind coxal sacs (arrows).

Morphometrics from the SEM images of 15 symphylans showed the following characters. **Size:** average symphylan 3.9 mm (range 2.9–4.75 mm excluding antennae). **Head:** somewhat heart-shaped, central rod had a knob before arriving to the posterior point of the head. Tömosvary organ was clearly defined with a hole in the centre (Figure 1A, B). **Antennae:** between 22 and 31 segments covered with setae (Figure 1A). **Abdomen:** scutes with pubescent cuticles, convex anterior tergites and last scuta margins with a U-shaped groove covered with thin dorsal setae, and long ventral and lateral setae (Figure 1C). **Legs:** presence of sternal appendages behind the 3rd to 9th coxal sacs (Figure 2) (Table 1).

**Table 1.** Morphological characters of the genera belonging to the family Scutigerellidae. Colombian symphylans share more than one of the characters that define known genera within Scutigerellidae as described by Domínguez (2009, 2010), Halliday (2004), and Naumann and Scheller (1977).

Genus	Head	Cuticle of the scutes	Anterior tergites	Abdomen: U-shape groove in the last scuta	Legs: sternal appendages behind coxal sacs	Biogeographical distribution
***Scutigerella***	Heart-shaped	Pubescent	Convex	Present	Absent	Subcosmopolitan, mainly in the northern temperate zones
***Hanseniella***	Rounded	Glabrous	Not convex	Absent	Absent	Subcosmopolitan, mainly Neotropical and warm temperate zones
***Millotellina***	Longer than broad	Pubescent	Not convex	Absent	Present	Africa, Madagascar, Reunión, Sri Lanka, New Guinea and Australia
***Scopoliella***	Rounded	Pubescent	Convex	Absent	Absent	North America
***Scolopendrelloides***	Heart-shaped	Glabrous	Not convex	Absent	Absent	South-East Asia and Australia
**Our specimens**	Heart-shaped	Pubescent	Not convex	Present	Present	Colombia

### Molecular analysis

Two out of ten symphylans captured from Antioquia were successfully characterised at *COI* (658 bp) and ITS2 (358 bp) and both specimens shared the same unique haplotypes for each marker. An open reading frame was read for *COI* indicating the sequence likely represented a functional protein-coding gene not a pseudogene. GenBank sequence accession numbers: KP696390-91 (*COI*) and KP696392-93 (ITS2).

A comparison of our *COI* symphylan haplotype with sequences deposited in GenBank showed low homology with: *Scutigerella causeyae* (77%, query cover 99%, GenBank DQ666065) and *Hanseniella* n. sp. (78%, query cover 92%, GenBank AF370839). Using BOLD Systems database, 100% sequence homology was found with six specimens from Cameroon, described as Phylum Arthropoda, class Symphyla, status private, 77% homology with *Scutigerella* sp. (N = 2) from Bavaria (status private), 77% with *Scutigerella causeyae* (N = 2) source locality unknown (status private) and 76% with *Scutigerella causeyae* from Austria, Salzburg (status private).

The ITS2 haplotype characterised from our symphylans showed low homology with a sequence of *Scutigerella* sp. (95%, query cover 62%, GenBank DQ666184) and *Hanseniella* sp. (91%, query cover 70%, GenBank AY210821). The ITS2 haplotype could not be compared using BOLD Systems as this database does not collect sequences for this molecular marker.

## Discussion

The taxonomy of the class Symphyla is unclear, a consequence of few published studies: two morphological keys for European (Edwards 1959a, b, Domínguez 2010) and one key for Neotropical (Scheller and Adis 1996) species. There are no published morphological descriptions or keys for Colombian Symphyla, therefore the exact number of genera and species is unknown. The only symphylan recorded in Colombia is *Scutigerella immaculata* (Agredo et al. 1988, Peña 1998, Corredor 1999), however, this species lacks formal morphological description and both the type specimen and the type locality (London, United Kingdom) have been destroyed and no redescription has been made (Scheller pers. comm.).

Our Colombian symphylans showed genus-level morphological ambiguity (Table 1). We observed a U-shaped groove in the anterior most scuta the character identifying *Scutigerella* (Halliday 2004), but paired sternal appendages behind the 3rd to 9th coxal sacs of the legs (Figure 2) that are unique to *Millotellina* (Naumann and Scheller 1977). Naumann and Scheller (1977) describe the sternal appendages in two subgenera of *Millotellina*, *Millotellina* with unpaired appendages between legs 5 and 10 and *Diplomillotellina* with pairs between legs 5 and 9. However, our symphylans presented paired appendages between legs 3 and 9, which could suggest the existence of a new subgenus within *Millotellina*.

According to Hebert et al. (2003) the threshold of genetic divergence for species delimitation is 3%. However, recent studies have shown that there is no single universal threshold for species’ delimitation using the barcode region, which can differ according to the group studied (Rach et al. 2008). For example, Ruiz et al. (2010, 2013) reported in mosquitoes of South America a lower interspecific threshold between 2 and 2.5%. To our knowledge only three papers have used *COI* bardcoding within the subphylum Myriapoda, to which class Symphyla belongs. Spelda et al. (2011) showed for class Chilopoda a mean interspecific genetic distance of 18.3%: range 12.0% between congeneric species to 25% between genera or families. Stoev et al. also for class Chilopoda showed mean interspecific genetic distances between 5 (2010) and 12 (2013) species of *Eupolybothrus* genus that ranged between 16.1–24.0% and 10.7–24.5%, respectively.

Our Colombian Symphyla *COI* haplotype showed genetic divergence with sequences of *Scutigerella causeyae* of at least 23% and *Hanseniella* n. sp. of 22%, similar to the congeneric ranges observed by Spelda et al. (2011) and Stoev et al. (2010, 2013). Unfortunately there are no published sequences of *Scutigerella immaculata* or a formal description of this species. As our specimens showed a mixture of morphological characters of *Scutigerella immaculata* and *Millotellina* genus, which has never before been reported in the literature, we speculate that Colombian symphylans belong to a new taxon. It is therefore necessary that a formal redescription of *Scutigerella immaculata* be published before the taxonomic status of these Colombian symphylans can be made.

It is interesting that our *COI* barcode shared the same haplotype as six Symphyla specimens found in Cameroon. This demonstrates that this taxon is not restricted to South America, it has a wide geographical distribution and therefore can be a wide-spread agricultural pest. We have two hypotheses to explain this taxon’s distribution: 1. That the specimens found in Colombia are a “tramp species”, which was introduced inadvertently by human commerce from Africa to the Americas or vice versa. 2. This taxon is native to Colombia, but due to the lack of specialists on this group along with the lack of morphological keys, this taxon has remained unrecognised.

## Conclusion

We demonstrate for class Symphyla that the parallel use of DNA barcoding with morphological descriptions can contribute to the taxonomic resolution of this understudied group. Our specimens presented not only the morphological characters of the only symphylan species reported in Colombia, *Scutigerella immaculata*, but also the character identifying species within *Millotellina* genus whose distribution has not been recorded in the Americas (Table 1). Furthermore, we showed the same Symphyla *COI* haplotype in both South America and Africa. This research highlights the need for further studies of morphology and molecular phylogenies that include type material to determine the worldwide taxonomic status of class Symphyla.

## References

[B1] Agredo C Zuluaga J Chaparro E (1988) Observaciones sobre características, distribución y daños de sinfílidos (Symphyla) y otros organismos del suelo, en cultivos de piña (Ananas comosus) del Valle del Cauca. Acta Agronómica 38: 65–73.

[B2] Collins F Paskewitz S (1996) A review of the use of ribosomal DNA (rDNA) to differentiate among cryptic Anopheles species. Insect Molecular Biology 5: 1–9. doi: 10.1111/j.1365-2583.1996.tb00034.x 8630529 10.1111/j.1365-2583.1996.tb00034.x

[B3] Corredor D (1999) Integrated pest management in cut flower crops grown in plastic houses at the Bogota Plateau. Acta Horticulturae 482: 241–246.

[B4] de Morais JW da Silva EP (2009) Occurrence of Symphyla (Myriapoda) in the region of the Upper Solimões River, Amazonas, Brazil. Pesquisa Agropecuária Brasileira 44: 981–983. doi: 10.1590/S0100-204X2009000800028

[B5] Domínguez MT (1992) Symphyla y Pauropoda (Myriapoda) de suelos de España II. Tesis Doctoral Universidad Complutense de Madrid.

[B6] Domínguez CM (2009) Phylogeny of the Symphyla (Myriapoda). PhD thesis, Freie University, Berlin.

[B7] Domínguez CM (2010) New insights on the genus Scolopendrelloides Bagnall 1913 (Scutigerellidae, Symphyla) with descriptions of two new species. Zootaxa 2558: 48–60.

[B8] Duran D (1982) Manejo de insectos y otros artrópodos relacionados con el cultivo de flores. Seminario de plagas en cultivos de flores. Sociedad Colombiana de Entomología. Bogotá, Colombia, 84–87.

[B9] Edgecombe GD (2004) Morphological data, extant Myriapoda, and the myriapod stem-group. Contributions to Zoology 73: 207–252.

[B10] Edwards CAT (1959a) A revision of the British Symphyla. Proceedings of the Zoological Society of London 132: 403–439. doi: 10.1111/j.1469-7998.1959.tb05529.x

[B11] Edwards CAT (1959b) Keys to the genera of the Symphyla. Journal of the Linnean Society of London (Zoology) 44: 164–169. doi: 10.1111/j.1096-3642.1959.tb01603.x

[B12] Folmer O Black M Hoeh W Lutz R Vrijenhoek R (1994) DNA primers for amplification of mitochondrial Cytochrome C Oxidase subunit I from diverse metazoan invertebrates. Molecular Marine Biology and Biotechnology 3: 294–299. 7881515

[B13] Halliday R (2004) Confirmation of the presence of *Scutigerella immaculata* (Newport) in Australia (Symphyla: Scutigerellidae). Australian Journal of Entomology 43: 43–45. doi: 10.1111/j.1440-6055.2003.00375.x

[B14] Hebert P (2003) Biological identifications through DNA barcodes. Proceedings of the Royal Society of London, Series B 270: 313–321. doi: 10.1098/rspb.2002.2218 12614582 10.1098/rspb.2002.2218 PMC1691236

[B15] Katoh K Misawa K Kumar K Miyata T (2002) MAFFT: a novel method for rapid multiple sequence alignment based on fast Fourier transform. Nucleic Acids Research 30: 3059–3066. doi: 10.1093/nar/gkf436 12136088 10.1093/nar/gkf436 PMC135756

[B16] Linton Y Harbach R Anthony T Chang M Asmad M (2001) Morphological and molecular identity of Anopheles (Cellia) sundaicus (Diptera: Culicidae), the nominotypical member of a malaria vector species complex in Southeast Asia. Systematic Entomology 26: 357–366. doi: 10.1046/j.1365-3113.2001.00153.x

[B17] Maddison DR Maddison WR (2003) MacClade v.4.06: analysis of phylogeny and character evolution. Sinauer Associates, Sunderland, MA. 10.1159/000156416 2606395

[B18] Mallatt J Garey J Shultz J (2004) Ecdysozoan phylogeny and Bayesian inference: first use of nearly complete 28S and 18S rRNA gene sequences to classify the arthropods and their kin. Molecular Phylogenetics and Evolution 31: 178–191. doi: 10.1016/j.ympev.2003.07.013 15019618 10.1016/j.ympev.2003.07.013

[B19] Michelbacher A (1938) Seasonal variation in the distribution of two species of Symphyla from California. Journal Economic Entomology 32: 53–57. doi: 10.1093/jee/32.1.53

[B20] Nauman D Scheller UF (1977) The genus *Millotellina* Jupeau in Australia (Myriapoda: Symphyla: Scutigerellidae). Journal of the Australian Entomological Society 16: 47–57. doi: 10.1111/j.1440-6055.1977.tb00059.x

[B21] Navarro R Gaviria B (2001) Resistencia de variedades de crisantemo a la pudrición de raíces (*Cylindrocarpon. destructans.* Zinssin) Schalten. Reinfestación por nematodos en suelos y nematofauna asociada a las aguas de riego. Serie de investigación, Asociación Colombiana de Exportadores de Flores ASOCOLFLORES, Universidad católica de Oriente, Unidad de Sanidad Vegetal, Rionegro, Antioquia, 30–34.

[B22] Padial J de La Riva I (2007) Integrative taxonomists should use and produce DNA Barcodes. Zootaxa 1586: 67–68.

[B23] Peña BC (1998) Evaluación de daños de *Scutigerella immaculata* (symphilia: scutigerellidae) en las primeras etapas de crecimiento y desarrollo de Clavel, Rosa y Pompón y su relación con el tipo de suelo. Tesis, Universidad Nacional de Colombia, Bogotá, Colombia.

[B24] Podsiadlowski L Kohlhagen H Koch M (2007) The complete mitochondrial genome of *Scutigerella causeyae* (Myriapoda: Symphyla) and the phylogenetic position of Symphyla. Molecular phylogenetics and evolution 45(1): 251–60. doi: 10.1016/j.ympev.2007.07.017 17764978 10.1016/j.ympev.2007.07.017

[B25] Rach J DeSalle R Sarkar IN Schierwater B Hadrys H (2008) Character-based DNA barcoding allows discrimination of genera, species and populations in Odonata. Proceedings of The Royal Society B. Biological Science 275: 237–247. doi: 10.1098/rspb.2007.1290 10.1098/rspb.2007.1290 PMC2212734 17999953

[B26] Ruiz F Linton Y Ponsonby D Conn J Herrera M Quiñones M Vélez I (2010) Molecular comparison of topotypic specimens confirms Anopheles (Nyssorhynchus) dunhami Causey (Diptera: Culicidae) in the Colombian Amazon. Memorias do Instituto Oswaldo Cruz 105: 899–903. 21120360 10.1590/s0074-02762010000700010 PMC4438775

[B27] Ruiz F Quiñones ML Erazo HF Calle DA Alzate JF Linton Y-M (2005) Molecular differentiation of Anopheles (Nyssorhynchus) benarrochi and An. (N.) oswaldoi from Southern Colombia. Memorias do Instituto Oswaldo Cruz 100: 155–160. doi: 10.1590/S0074-02762005000200008 16021302 10.1590/s0074-02762005000200008

[B28] Ruiz F Wilkerson R Ponsonby D Herrera M Sallum MA Velez I Quiñones M Velez I Florez-Mendoza C Alarcon J Alarcon-Ormasa J Liton Y-M (2013) Systematics of the oswaldoi complex (*Anopheles*, *Nyssorhynchus*) in South America. Parasites and Vectors 6: . doi: 10.1186/1756-3305-6-324 10.1186/1756-3305-6-324 PMC3843595 24499562

[B29] Scheller U (1961) A review of the Australian Symphyla (Myriapoda). Australian Journal of Zoology 9: 140–171. doi: 10.1071/ZO9610140

[B30] Scheller U (1982) Synopsis and classification of living organisms. In: Parker SP (Ed.) McGraw Hill, New York, 688–689.

[B31] Scheller U Adis J (1996) A Pictorial Key for the Symphylan Families and Genera of the Neotropical Region South of Central Mexico (Myriapoda, Symphyla). Studies on Neotropical Fauna and Environment 31: 57–61. doi: 10.1076/snfe.31.1.57.13316

[B32] Shear WA Edgecombe GD (2010) The geological record and phylogeny of the Myriapoda. Arthropod Structure and Development 39: 174–190. doi: 10.1016/j.asd.2009.11.002 19944188 10.1016/j.asd.2009.11.002

[B33] Smith MA Fisher BL Hebert PDN (2005) DNA barcoding for effective biodiversity assessment of a hyperdiverse arthropod group: the ants of Madagascar. Philosophical Transactions of the Royal Society B: Biological Sciences 360: 1825–1834. doi: 10.1098/rstb.2005.1714 10.1098/rstb.2005.1714 PMC1609228 16214741

[B34] Spelda J Reip HS Oliveira-Biener U Roland RM (2011) Barcoding Fauna Bavarica: Myriapoda – a contribution to DNA sequence-based identifications of centipedes and millipedes (Chilopoda, Diplopoda). ZooKeys 156: 123–139. doi: 10.3897/zookeys.156.2176 22303099 10.3897/zookeys.156.2176 PMC3253575

[B35] Stoev P Akkari N Zapparoli M Porco D Enghoff H Edgecombe GD Georgiev T Penev L (2010) The centipede genus *Eupolybothrus* Verhoeff, 1907 (Chilopoda: Lithobiomorpha: Lithobiidae) in North Africa, a cybertaxonomic revision, with a key to all species in the genus and the first use of DNA barcoding for the group. ZooKeys 50: 29–77. doi: 10.3897/zookeys.50.504 21594115 10.3897/zookeys.50.504 PMC3088018

[B36] Stoev P Komericki A Akkari N Liu S Zhou X Weigand AM Hostens J Hunter CI Edmunds SC Porco D Zapparoli M Georgiev T Mietchen D Roberts D Faulwetter S Smith V Penev L (2013) *Eupolybothrus cavernicolus* Komericki and Stoev sp. n. (Chilopoda: Lithobiomorpha: Lithobiidae): the first eukaryotic species description combining transcriptomic, DNA barcoding and micro-CT imaging data. Biodiversity Data Journal 1: . doi: 10.3897/BDJ.1.e1013 10.3897/BDJ.1.e1013 PMC3964625 24723752

[B37] Umble J Dufour R Fisher G Leap J Van Horn M (2006) Symphylans: soil pest management options. National Sustainable Agriculture Information Service, National Center for Appropriate Technology. Available online.

[B38] Wiemers M Keller A Wolf M (2009) ITS2 secondary structure improves phylogeny estimation in a radiation of blue butterflies of the subgenus Agrodiaetus (Lepidoptera: Lycaenidae: Polymmatus). BMC Evolutionary Biology 9: . doi: 10.1186/1471-2148-9-300 10.1186/1471-2148-9-300 PMC2810301 20035628

